# South Africa field epidemiology training program: developing and building applied epidemiology capacity, 2007–2016

**DOI:** 10.1186/s12889-019-6788-z

**Published:** 2019-05-10

**Authors:** Carl Reddy, Lazarus Kuonza, Hetani Ngobeni, Natalie T. Mayet, Timothy J. Doyle, Seymour Williams

**Affiliations:** 1 National Institute for Communicable Diseases, National Health Laboratory Service, Johannesburg, South Africa; 2 Center for Global Health, Centers for Disease Control and Prevention, Pretoria, South Africa

## Abstract

In 2007, South Africa (SA) launched a field epidemiology training program (SAFETP) to enhance its capacity to prevent, detect, and respond to public health threats through training in field epidemiology. The SAFETP began as a collaboration between the SA National Department of Health (NDOH), National Institute for Communicable Diseases (NICD), and the University of Pretoria (UP), with technical and financial support from the U.S. Centers for Disease Control and Prevention (CDC). In 2010, the CDC in collaboration with the NICD, established a Global Disease Detection (GDD) Center in SA, and the SAFETP became a core activity of the GDD center. Similar to other FETPs globally, the SAFETP is a 2-year, competency-based, applied epidemiology training program, following an apprenticeship model of ‘learn by doing’. SAFETP residents spend approximately 25% of the training in classroom-based didactic learning activities, and 75% in field activities to attain core competencies in epidemiology, biostatistics, outbreak investigation, scientific communication, surveillance evaluation, teaching others, and public health leadership. Residents earn a Master’s in Public Health (MPH) degree from UP upon successfully completing a planned research study that serves as a mini-dissertation.

Since 2007, SAFETP has enrolled an average of 10 residents each year and, in 2017, enrolled its 11th cohort. During the first 10 years of the program, 98 residents have been enrolled, 89% completed the 2-year program, and of these, 76 (87%) earned an MPH degree. Of those completing the program, 88% are employed in the public health sector, and work at NICD, NDOH, Provincial Health Departments, foreign health institutions, or non-governmental organizations. In the first 10 years of the program, the combined outputs of trainees included over 130 outbreak investigations, more than 150 abstracts presented at national and international scientific conferences, more than 80 surveillance system evaluations, and more than 45 manuscripts published in peer-reviewed scientific journals. The SAFETP is having an impact in building epidemiology capacity for public health in South Africa. Developing methods to directly link and measure the impact of the program is planned for the future.

## Background

There is increasing worldwide recognition that having applied epidemiology training that leads to the development of skilled personnel who conduct surveillance, investigate outbreaks, estimate disease burden, and evaluate determinants of health and the impacts of public health interventions is essential for every country. [1] This workforce contributes to an effective and high quality public health system. [2] South Africa has recognized the importance of human resource development as it commits to the re-engineering of primary health care, and moves to provide universal access to health care for all South Africans. [3] Based on international recommendations of at least 1 field epidemiologist per 200,000 population, [1] the country requires at least 275 field epidemiologists. However, a national epidemiology capacity assessment has not been done. [4] Therefore, it is uncertain how many epidemiologists there are in the country and there is limited information on the number of staff who perform specific epidemiological functions at different levels of the public health system.

Starting in 2007, the South Africa Field Epidemiology Training Program (SAFETP), a 2-year, competency-based training program, was initiated and developed as a collaboration between the South African National Department of Health (NDOH), the National Institute of Communicable Diseases (NICD) of the National Health Laboratory Service (NHLS), and the U.S. Centers for Disease Control and Prevention (CDC), to increase field epidemiology capacity for the country. SAFETP uses an established applied epidemiology curriculum, providing an accredited Masters in Public Health (MPH) degree from the University of Pretoria (UP), with mentored competency-based practical field experience. To date, the program has trained 87 health professionals in applied epidemiology. This paper describes the first 10 years of the SAFETP, including the origin and rationale for the program, the training model, accomplishments, challenges encountered, and observations regarding possible future directions of the program.

### Origin, genesis, and evolution of SAFETP

Training in applied field epidemiology at a national level has its origins in the 1950’s with the creation of the Epidemic Intelligence Service (EIS) at CDC. [5, 6] The creators of EIS envisioned a competency-based training program in applied epidemiology modeled after a medical residency, in which participants learn by doing, under the close guidance and supervision of an experienced mentor. EIS has never been an academic degree awarding program, but rather a post-doctoral training program designed to develop the next generation of trained field epidemiologists to serve the U.S. public health system. During the first 63 years of the program, EIS has trained 3641 health professionals in applied epidemiology (personal communication, EIS program office).

Beginning in the 1970’s, the applied epidemiology training model of EIS was exported to other countries, often under the name of Field Epidemiology Training Programs (FETP). [7–9] Early FETPs were consistent with EIS, in that they were non-academic, non-degree granting programs. In some regions of the world, including Africa, it was recognized that gaining academic credentials, particularly in a lengthy 2-year training activity, was a powerful incentive for participants in the training. Therefore, some training programs partnered with universities to offer master’s degrees in conjunction with the training. In Africa, the earliest FETP-like training programs began in Uganda and Zimbabwe and were affiliated with academic institutions which awarded participants a master’s degree following successful completion of the training. [10] These programs were referred to as “*Public Health Schools Without Walls”* (PHSWOW), to convey both the academic and the applied field components of the training.

Beginning in the 2000’s, many other African countries recognized the need for human resource development, not only in epidemiology, but also in laboratory sciences linked to applied epidemiology. During this decade, many African nations hardest hit by the HIV/AIDS pandemic also began receiving substantial resources to combat HIV, and develop the human resources necessary to address the epidemic, from the U.S. President’s Emergency Plan for AIDS Relief (PEPFAR) and its predecessor, the LIFE initiative. In this context, training programs that included content in both field epidemiology and laboratory services were launched in Kenya and Tanzania in 2004–2005, and referred to as Field Epidemiology and Laboratory Training Programs or FELTP. These programs closely resembled the standard FETPs outside of Africa, but included a laboratory component, and were also affiliated with academic institutions awarding a master’s degree, similar to the PHSWOW programs in Uganda and Zimbabwe. These FELTP’s in Kenya and Tanzania quickly became the model for establishing other FELTP’s in Africa. [11] While FETPs have traditionally targeted physicians, nurses, and surveillance officers, FELTPs have additionally enrolled laboratory scientists in the training.

Following establishment of democratic rule in South Africa in the early 1990’s, the country was faced with growing expectations to provide basic health services for all, in the context of substantial racial disparities in economic and health status. The country also reported one of the highest numbers in the world of people living with HIV, high rates of TB and other infectious diseases, as well as a growing chronic disease burden. [12] In this context, the South African National Department of Health recognized the growing need to improve surveillance systems and the need to build epidemiology capacity.

In 2004, South Africa became one of the 15 PEPFAR focus countries and received the largest PEPFAR budget. The need to build applied epidemiology capacity in South Africa was prioritized to effectively respond to its HIV/AIDS epidemic. In 2005, South African health officials, together with U.S. and locally based CDC officials, planned the development of a South Africa Field Epidemiology and Laboratory Training Program (SAFELTP), which was subsequently launched in 2007. SAFELTP began as an academic degree granting program with a laboratory training component, similar to the Kenya and Tanzanian FELTPs, and supported as a collaboration between the NDOH, NICD, and UP School of Health Systems and Public Health, with technical and financial assistance from U.S. CDC and PEPFAR. The program targeted physicians, nurses, laboratorians, and other allied health professionals with a first degree in the health sciences. Other details regarding the early origins of the program have been previously described. [13]

In 2012, the program conducted an external review (unpublished) and the outcome of the review informed the strategic plan of the program. The sustainability of the program, together with increasing its visibility and impact, were listed as key strategic objectives. Moreover, demand for the laboratory component of the program diminished over time and it was observed that laboratory scientists enrolled in FELTP did not return to work in the laboratories following conclusion of the program. In view of this, the laboratory component was removed in 2014, thus the SAFELTP became the SAFETP it is today, and no longer includes a separate laboratory training track.

Another major transition occurring during the first 10 years relates to funding for the program. When the program began, the majority of the operating costs were from direct assistance via PEPFAR. Additional resources were provided by the South African NDOH through its allocation of funds to the NICD of the NHLS. In 2010, CDC in collaboration with the NICD, established a Global Disease Detection (GDD) center in South Africa. The GDD center was established as a partnership to improve early detection of health threats that could be regional or global in nature, similar to the 9 other CDC supported GDD centers around the world. [14] Over time, the financial support for the SAFELTP, and subsequently the SAFETP, transitioned from primarily PEPFAR support, to a greater proportion of financial support provided by GDD. By 2015, GDD provided all U.S. government financial support. Also during this period, an increasing proportion of SAFETP costs were covered by the NICD host institution. As of 2016, all direct program costs are covered by NICD, while CDC GDD supports some technical assistance costs.

### Training model

Participants in the SAFETP develop competencies in several inter-related areas: epidemiology, disease surveillance, biostatistics, data management and analysis, scientific communication, public health leadership, and teaching of others. Residents develop these competencies by elaborating a set of Core Learning Activities (CLAs). The CLAs required to complete the program include: 1) evaluating a surveillance system; 2) analyzing a large data set; 3) developing a protocol for and completing a hypothesis driven research project; 4) submitting abstracts for scientific conferences; 5) a poster or oral presentation at a scientific conference; 6) preparing a bulletin article on a public health topic; 7) preparing a scientific manuscript suitable for publication in a peer-reviewed journal; 8) investigating at least 3 disease outbreaks; 9) conducting workshops or other training activities for others. While developing these CLAs, related protocols, and work products, residents are also trained in the core ethical principles of conducting research involving human subjects. Residents must complete all required CLAs to be eligible for a UP degree. In addition, they must complete a standard academic thesis or a mini-dissertation. For their theses, residents are encouraged to prepare a manuscript formatted as a peer-reviewed journal article, based on one of their CLAs.

The SAFETP enrolls a new cohort of residents annually that begin their 2-year training in January, and complete the training in December of the following year. During the 24 months of the program, residents spend the first 5 months in classroom modules at UP or NICD, to gain knowledge and skills needed to develop the required CLAs. In June of each year, the first year residents are then assigned to their field locations and field site supervisor. During the final 18 months of the program, residents develop and complete their CLAs, with assistance from their field supervisor and program mentor.

Residents spend approximately 75% of the 2-year training at field placement sites. These sites are selected based on priority health areas where adequate supervision, a suitable work environment, and access to data and other resources are available for completing the CLAs. Residents focus on addressing the priority diseases of the country (e.g. HIV, TB, respiratory illnesses, enteric diseases, or chronic diseases). In the past, residents have been placed at the National Department of Health, the National Cancer Registry, the University of South Africa (UNISA) Injury and Violence Unit, Health Sciences Research Council (HSRC), and Medical University of South Africa (MEDUNSA) HIV Pharmacovigilance Unit. Other specific field sites may include the NICD, a provincial or municipal health department, or partner ministries such as the Department of Agriculture, Forestry, and Fisheries (DAFF), which is responsible for animal health and many zoonotic disease issues. Field sites interested in hosting a SAFETP resident complete an application form and describe the data and resources they have available to host a resident. Decisions regarding final field placements consider adequacy of data and supervision, local knowledge and language skills of the resident, and priority health areas.

The NICD has an Outbreak Response Unit (ORU) staffed by a small number of field epidemiologists who assist provinces and districts in responding to and investigating disease outbreaks. Each SAFETP resident completes a 3–4 week rotation at ORU during their training. The rotation provides supervised opportunities to develop needed knowledge and skills in investigating disease outbreaks. Residents may also complete outbreak investigations during their regular field assignments.

Other important components of the training program include a quarterly scientific seminar in which the residents present findings from field investigations; participation at national and international scientific conferences to present accepted work; and supplemental workshops on relevant topics.

For completion of the CLAs and UP thesis requirements, residents are supported by a SAFETP assigned mentor and a field supervisor. In addition, GDD provided technical assistance has included a scientific writer, a biostatistician, and a CDC FETP resident adviser, who have provided training and support to all residents in scientific communication, statistical analysis, and epidemiologic field investigation methods. The scientific writer conducts workshops on scientific communication which address the specific requirements of each of the written CLAs. The writer-editor reviews draft written materials and provides one-on-one consultation during the scientific writing process. The biostatistician provides individual consultation regarding survey design, sample size calculation, and statistical analysis.

SAFETP has also been an active member of the relevant professional networks for training in field epidemiology. This includes at the global level, the Training Programs in Epidemiology and Public Health Interventions Network (TEPHINET), [15] and regionally in Africa, the African Field Epidemiology Network (AFENET). [10] Membership and participation in these professional networks has enhanced training activities for residents via training workshops and opportunities to present at TEPHINET and AFENET sponsored international scientific conferences. The networks facilitate sharing of training materials and provide standards for accreditation of FETPs. The SAFETP Program Director has served on the advisory boards of TEPHINET and AFENET, thus leveraging the experience of SAFETP to enhance similar training activities in other countries.

### Program accomplishments

The total number of residents enrolled in SAFETP between 2007 and 2016 is 98 (average 10 residents/year). Of these, 87 (89%) have completed the two-years of residency and of these 87, 76 (87%) have been awarded MPH degrees by the University of Pretoria (as at May 2018) (Table 1). Although most SAFETP enrollees are South Africans, the program has also enrolled and graduated foreign residents from Burkina Faso (1), Togo (1) and Liberia (2). The incoming class of 2017 also includes one resident from Lesotho.

**Table 1:** South Africa Field Epidemiology Training Program enrollment and completion rate, 2007–2016

Enrollment year	Number enrolled	Completion	
		Two-year course, n (%)	MPH Degree, n (%^a^)
2007	10	9 (90)	8 (89)
2008	9	9 (100)	9 (100)
2009	12	11 (92)	9 (82)
2010	11	9 (82)	9 (100)
2011	9	9 (100)	7 (78)
2012	5	5 (100)	5 (100)
2013	9	6 (67)	4 (67)
2014	15	13 (87)	10 (77)
2015	11	9 (82)	8 (89)
2016	7	7 (100)	7 (100)
Total	98	87 (89)	76 (87)

^a^As proportion of those completing two-year course

### Sectors where graduates are employed

Of the 66 SAFETP graduates who earned MPH degrees and were employed as of May 2017, 59 (89%) are working within South Africa (Table 2). Of the seven that are working outside of South Africa, four are the foreign graduates who work in public health positions in their home countries, two are continuing public health training abroad, and one is working at the Africa Centers for Disease Control and Prevention (Africa CDC) based at the African Union Commission in Ethiopia. Of 59 graduates working in SA, 58% are working for South African government institutions, either at the National (32%) or Provincial (26%) level. In addition, 18 (27%) are working for non-governmental organizations (NGOs), which are more concentrated in Gauteng Province.

**Table 2:** Employment of MPH graduates of the South Africa Field Epidemiology Training Program

Location	MPH Graduates		Population*	FETP Trained Epidemiologists
	n	%	(in millions)	(per 200,000 population)
South Africa	59	89	55.6	0.212
National-Governmental	21	32	55.6	0.076
Provincial Health Agency - All	17	26	NA	
Northern Cape	2	3	1.2	0.333
Limpopo	4	6	5.8	0.138
Eastern Cape	4	6	7.0	0.114
Free State	1	1.5	2.8	0.071
North West	1	1.5	3.7	0.054
Mpumalanga	1	1.5	4.3	0.047
Western Cape	1	1.5	6.3	0.032
Gauteng	2	3	13.4	0.030
KwaZulu-Natal	1	1.5	11.1	0.018
Subtotal: National or Provincial Health Agency	38	58	55.6	0.137
NGO	18	27	NA	
Academia	3	5	NA	
Outside South Africa	7	11	NA	
Total	66**	100		

*NGO* Non-governmental Organization

*2016 populations estimates from Statistics South Africa (http://www.statssa.gov.za/)

**Includes only employed MPH graduates as of May 2017

The number of SAFETP graduates per 200,000 population was calculated nationally and by province of employment (Table 2). Overall, SAFETP graduates working in South Africa account for 0.212 epidemiologists per 200,000 population. When excluding those working for NGOs or in academic settings, and counting only the 38 graduates working in applied epidemiology positions at the national or provincial level, SAFETP graduates represented 0.137 field epidemiologists per 200,000 population. Though both estimates fall short of the 1 field epidemiologist per 200,000 population suggested by the WHO Joint External Evaluation Tool, [1] it is important to keep in mind that SAFETP is not the only source of epidemiologists working in the country, and that a full capacity assessment in South Africa has not been performed.

### Program impact

A comprehensive formal impact evaluation of the SAFETP has never been conducted, however, evidence of the impact can be gleaned from the work produced by the residents during their training, its impact on the health system as a whole, and the final disposition of graduates in key positions. [16] A formal evaluation to measure the impact of SAFETP is underway and complete findings are not yet available. However, some examples of the programs impact are noted below.

Over 45 scientific manuscripts have been published in peer-reviewed scientific journals by SAFETP residents, based on their activities during the training program. [17] SAFETP trainees have made significant contributions in investigating and responding to numerous disease outbreaks and priority health conditions and control activities throughout the country that have been key inputs for local, provincial, and national public health decision making (Table 3). Selected examples of the outbreaks include outbreaks of Rift Valley fever during 2008–2011 that led to greater collaborations with the veterinary and animal health sector; [18, 19] an outbreak of multi-drug resistant *Pseudomonas aeruginosa* in a hospital in Cape Town in 2010; [20] a diarrheal disease outbreak investigation in Free State that led to major improvements in the water treatment system; and, identifying the emergence of multi-drug resistant *Candida auris* fungal infections, leading to the development of clinical and diagnostic guidelines for detection and investigation of future outbreaks. [21, 22] SAFETP residents have also responded to outbreaks in other African countries in the region. Selected examples of these include an outbreak of typhoid in Zimbabwe in 2011–12, [23] which highlighted the role of contaminated boreholes in continued transmission; an outbreak of diarrheal illness in Swaziland in 2014; and an investigation of a cluster of hydrocephalus cases in Lesotho in 2017. Finally, residents have completed analytic research studies on the determinants of loss to follow-up among HIV patients in South Africa on anti-retroviral treatment enrolled in a pharmacovigilance cohort study from 2004 to 2012. [24] The results bolstered the strategy of community-based adherence support programs and integrated patient care, implemented by the South African HIV/AIDS treatment program. Residents also conducted analyses identifying risk factors for tuberculosis smear non-conversion in Western Cape Province from 2007 to 2013, [25] helping health providers better target at-risk patients.

**Table 3:** Summary of key activities of the South Africa Field Epidemiology Training Program, 2007–2016

Activity	Frequency
Outbreak investigations	> 130
Surveillance evaluations	> 80
Planned research studies	> 50
Large data base analysis	> 85
Scientific presentations at national or international conferences	> 150
Awards received by trainees at scientific conferences	11
Publications in peer reviewed journals	> 45

SAFETP trainees and graduates have also helped strengthen public health surveillance programs within the country. The trainees have contributed to the design, implementation, and evaluation of various surveillance systems at all levels of the health system. During 2015, a team of SAFETP residents (*n* = 6) and alumni (*n* = 7) conducted an evaluation of the National Notifiable Medical Conditions (NMC) surveillance system in the country. The findings from the evaluation have informed the recent re-engineering of the NMC surveillance system for South Africa.

### Challenges

SAFETP has experienced several challenges over its first decade. The external program review in 2012 confirmed that at least 30% of residents had completed all requirements except the UP thesis, and thus, were not graduating with their MPH on time. UP had always offered a peer-reviewed manuscript as an option instead of a traditional academic thesis. However, the majority of residents had been submitting the traditional academic thesis. The SAFETP adopted the external reviewers’ recommendation and made a first authored, peer-reviewed manuscript compulsory and encouraged residents to use this manuscript as the basis for fulfilling the UP thesis requirement for graduation. SAFETP staff then followed up with individual residents who had previously completed the two year training, but had not achieved the MPH. This involved one-on-one meetings to review their progress in manuscript writing, statistical analysis, and interpretation of their data. As a result, the overall MPH graduation rate went up from 55%, as measured in 2013, to 87% measured in 2018, following the completion of the 2016 cohort.

The external evaluation also pointed out that the program was not attracting medical or veterinary graduates for enrollment. Less than 20% of the program participants to date have been medical doctors or veterinarians. Ongoing efforts are underway, working with the leadership of NDOH and DAFF, to facilitate and support more medical and veterinary staff from those agencies to join SAFETP. DAFF is supporting two veterinarians currently in the program. The demand for curative care in South Africa places undue pressure on medical doctors to go into providing clinical services. However, supervisory staff at facility and provincial DOH levels have shown willingness to support nurses and medical scientists to join the program. Fully addressing this issue is an ongoing challenge, however, SAFETP has recently undertaken a series of focus group discussions on field epidemiology for fourth-year medical and veterinary students to increase awareness of the opportunities that the discipline presents.

An ongoing program challenge is increasing the quantity and quality of outbreak investigations conducted by the residents. Residents are required to participate in at least three outbreak investigations. Their individual responsibilities in each investigation gradually increase, as their competency in outbreak investigation progresses, so that by the third outbreak, they are expected to lead the investigation. Residents may partially complete their outbreak investigation competency through the ORU. The number of requests, the type of investigations requested, along with ORU staff availability and standardization of the outbreak investigation approach, have been identified as factors leading to variable resident experiences in learning to do outbreak investigations. In general, residents assigned to provinces and districts have an increased likelihood of being involved in outbreak investigations within their jurisdiction. Ongoing efforts are underway to improve the coordination, supervision, and standardization of outbreak reporting and investigation, to better prepare SAFETP residents to acquire this critical competency.

### Future directions

#### Sustainability and transition to NICD funding support

SAFETP started with being almost completely funded by the US CDC. Since inception, the NICD has provided office space and other program services such as human resources, information technology, and grants management. In addition, the salary of the Program Director has always been funded by the NICD. A number of residents were financially supported by the NDOH while in the program through contribution of salaries and the provision of study leave. As of April 2016, the NICD became the primary funder of the program and the FETP posts of Senior Epidemiologist, Field Epidemiologist, and Administrator are now included in the NICD organogram staffing plan. Funding for the program comes from the South African Treasury through the NDOH to the NICD. The SAFETP’s funding transition to NICD brings country ownership and sustainability to the program.

#### Raising the profile of epidemiology in general and field epidemiology in particular

To facilitate the development and use of field epidemiology practice in the country, SAFETP staff has engaged in certain training and advocacy activities. To launch the program and since inception, SAFETP staff has provided short courses in field epidemiology to provinces and districts that have requested this training. These short courses have served to both market field epidemiology as a core public health discipline, and as a potential screening opportunity for identifying suitable candidates for the 2-year program. Since 2008, SAFETP has conducted approximately 20 basic epidemiology short courses, involving more than 500 participants, from all 9 provinces of South Africa. Recently, the CDC developed Frontline FETP training curriculum has been piloted in South Africa to further strengthen and enhance the existing training curriculum used for epidemiology short course training activities. [26] Future plans envision approximately 2–3 basic epidemiology short courses being conducted each year, for provincial and district level surveillance staff. Such courses also offer valuable opportunities for residents in the SAFETP 2-year course to develop competency in teaching principles of disease surveillance and applied epidemiology to others.

The human resources for health strategy document of the NDOH highlights the need to have a standardized, competency-based, and accredited training program for trainees in epidemiology and biostatistics, with specified career pathways and job opportunities following their training. [3] To this end, SAFETP program staff facilitated the establishment of an Epidemiology Working Group at the Public Health Association of South Africa (PHASA) to highlight the unmet need and support the development of applied epidemiology as a distinct career path. This working group will form part of the Epidemiology Special Interest Group (SIG) of PHASA and will help to define and build the career path for applied epidemiology in South Africa.

## Conclusions

From 2007 to 2016, SAFETP has matured from an externally supported collaboration, into a South African owned training program for developing applied epidemiologists to address the need for a quality public health system that is effective in detecting, responding to, and preventing health threats to its population. The leadership of SAFETP is forging the program into more formal engagement and deeper integration within the epidemiology and communicable diseases sections of the national and provincial departments of health. Additionally, to ensure that demand and that career pathways are specified, staffing plans with field epidemiology position descriptions must be further developed with principals at the National and Provincial DOH Human Resource Departments and NICD, in collaboration with leads from the Epidemiology SIG. In order to sustain field epidemiology training in the long term, the 10 years of SAFETP illustrates the need for a continued structured programmatic approach, including appropriate curriculum and mentored practicum sites that meet the priority public health conditions of the country.

## Abbreviations

AFENET: African Field Epidemiology Network
CDC: United States Centers for Disease Control and Prevention
CLA: Core Learning Activities
DAFF: Department of Agriculture, Forestry, and Fisheries
EIS: Epidemic Intelligence Service
FELTP: Field Epidemiology and Laboratory Training Program
FETP: Field Epidemiology Training Program
GDD: Global Disease Detection
HSRC: Health Sciences Research Council
MEDUSA: Medical University of South Africa
MPH: Master’s of Public Health
NDOH: South African National Department of Health
NGO: Non-governmental organization
NHLS: National Health Laboratory Service
NICD: National Institute of Communicable Diseases
NMC: Notifiable Medical Conditions
ORU: Outbreak Response Unit
PEPFAR: U.S. President’s Plan for Emergency AIDS Relief
PHASA: Public Health Association of South Africa
PWSWOW: Public Health School Without Walls
SAFETP: South African Field Epidemiology Training Program
SIG: Special Interest Group
TEPHINET: Training Programs in Epidemiology and Public Health Interventions Network
UNISA: University of South Africa
UP: University of Pretoria
